# COVID-19-Related Neuropsychiatric Symptoms in Patients With Alcohol Abuse Conditions During the SARS-CoV-2 Pandemic: A Retrospective Cohort Study Using Real World Data From Electronic Health Records of a Tertiary Hospital

**DOI:** 10.3389/fneur.2021.630566

**Published:** 2021-03-03

**Authors:** Carolina Varela Rodríguez, Francisco Arias Horcajadas, Cristina Martín-Arriscado Arroba, Carolina Combarro Ripoll, Alba Juanes Gonzalez, Marina Esperesate Pajares, Irene Rodrigo Holgado, Álvaro Cadenas Manceñido, Laura Sánchez Rodríguez, Blanca Baselga Penalva, Marta Marín, Gabriel Rubio

**Affiliations:** ^1^Quality of Care Unit, Hospital Universitario 12 de Octubre, Madrid, Spain; ^2^Psychiatric Department, Hospital Universitario 12 de Octubre, Madrid, Spain; ^3^RETIC (Network of Addictive Conditions), Institute of Health Carlos III, Madrid, Spain; ^4^Research and Science Support Unit, Instituto de investigación Biomédica del Hospital Universitario 12 de Octubre I+12, Madrid, Spain; ^5^Preventive Medicine Department, Hospital Universitario 12 de Octubre, Madrid, Spain; ^6^Preventive Medicine Department, Hospital General Nuestra Señora del Prado de Talavera, Toledo, Spain

**Keywords:** alcohol abuse (AA), COVID-19, neuropsychiatric symptoms, real world data, electronic health record

## Abstract

Patients with an alcohol abuse disorder exhibit several medical characteristics and social determinants, which suggest a greater vulnerability to the severe acute respiratory syndrome coronavirus 2 (SARS-CoV-2) infection and a worse course of the coronavirus disease 2019 (COVID-19) once infected. During the first wave of the COVID-19, most of the countries have register an increase in alcohol consumption. However, studies on the impact of alcohol addiction on the risk of COVID-19 infection are very scarce and inconclusive. This research offers a descriptive observational retrospective cohort study using real world data obtained from the Electronic Health Records. We found that patients with a personal history of alcohol abuse were 8% more likely to extend their hospitalization length of stay for 1 day (95% CI = 1.04–1.12) and 15% more likely to extend their Intensive Care Unit (ICU) length of stay (95% CI = 1.01–1.30). They were also 5.47 times more at risk of needing an ICU admission (95% CI = 1.61–18.57) and 3.54 times (95% CI = 1.51–8.30) more at risk of needing a respirator. Regarding COVID-19 symptoms, patients with a personal history of alcohol abuse were 91% more likely of exhibiting dyspnea (95% CI = 1.03–3.55) and 3.15 times more at risk of showing at least one neuropsychiatric symptom (95% CI = 1.61–6.17). In addition, they showed statistically significant differences in the number of neuropsychiatric symptoms developed during the COVID-19 infection. Therefore, we strongly recommend to warn of the negative consequences of alcohol abuse over COVID-19 complications. For this purpose. Clinicians should systematically assess history of alcohol issues and drinking habits in all patients, especially for those who seek medical advice regarding COVID-19 infection, in order to predict its severity of symptoms and potential complications. Moreover, this information should be included, in a structured field, into the Electronic Health Record to facilitate the automatic extraction of data, in real time, useful to evaluate the decision-making process in a dynamic context.

## Introduction

Patients with a substance abuse condition (SAC), such as alcoholic patients, exhibit several medical characteristicsm and social determinants that suggest a greater vulnerability to the severe acute respiratory syndrome coronavirus 2 (SARS-CoV-2) infection and, even more, a worse course of the coronavirus disease (2019) COVID-19 once infected.

According to the European Monitoring Center for Drugs and Drug Addiction and several studies (1, 2) on the implications of COVID-19 for illegal drugs users, they may suffer from more cardiovascular and respiratory pathologies, being also more vulnerable to damage from COVID-19 infection (3, 4). In addition to this cardiopulmonary comorbidity, the fact that immune response may be compromised in this population and that they may lack health-seeking behavior could increase the severity of the COVID-19 infection (5). Moreover, medications used for COVID-19 may be less effective and worse tolerated by drug users, who are also at an increased risk for pharmacological agent–drug of abuse interactions (6, 7).

An independent effect of “chronic alcohol abuse” on acute respiratory distress syndrome (ARDS) in critically ill patients has been demonstrated in a prospective cohort study (8). A recent systematic review and meta-analysis found that any measure of high relative to low alcohol consumption was associated with a significantly increased risk of acute respiratory distress syndrome (ARDS) [odds ratio (OR) 1.89; 95% CI, 1.45–2.48] (9). Alcohol can increase the risk of developing ARDS through various mechanisms including alveolar epithelium dysfunction, alcohol-induced oxidative stress, and interference on alveolar macrophage function (9). In hospitalized patients with pneumonia, having an alcohol-related diagnosis was associated with a greater likelihood of admission to the Intensive Care Unit (ICU) (OR, 1.63) and a longer length of stay (adding extra 0.6 days) (10).

Although an increase in alcohol consumption has been a constant in most of the countries that have gone through the first wave of COVID-19 (1), studies on the impact of alcohol addiction on the risk of COVID-19 infection are very scarce and inconclusive (11). In the 760 cases of COVID-19 detected in a cohort of 387,109 adults followed up in the United Kingdom, obesity and smoking increased the risk of infection but not heavy drinking, although the impact of alcohol abuse was not studied (11). Understanding heavy drinking for men as consuming 15 drinks or more per week, and for women 8 drinks or more per week, the average profile of patients with an alcohol abuse condition attended at the Hospital 12 de Octubre is of a heavy drinker with a daily basis and a systematic alcohol consumption. It is worth to note that the binge drinking is usual in Spain, but the person does not usually seek medical advice neither at the hospital nor at the Primary Care System; since asking for toxic habits is not systematically explored during the medical intervention unless directly related medical issues related to them are observed, these patients have no alcohol abuse condition diagnosis noted in their medical records.

Considering that alcohol-dependent patients associate multiple medical comorbidities and immune system disorders, in both phases, active consumption and withdrawal, it is foreseeable to assume that this population is more prone to get infected and suffer more severe complications from COVID-19 (2, 12). Hence, our aim is to assess whether alcohol consumption is associated with an increased severity of COVID-19 infection in hospitalized patients and an increased presence of neurological symptoms, considering the co-occurrence of other possible medical comorbidities.

## Materials and Methods

### Study Design

Descriptive, observational, retrospective cohort study in which data were obtained from Electronic Health Records (EHRs), admission information, and *ad-hoc* fulfillment of some variables of interest. The inclusion criteria were as follows: having at least one episode of hospitalization at the Hospital Universitario 12 de Octubre between February 25th and September 4th, 2020 and a diagnosis of COVID-19. The exposition criterion was having at least one diagnosis related to past or present alcoholism. The exclusion criteria were as follows: patients with incomplete data and patients with confidential occupational health data or errors due to low data quality. A match was performed using the propensity score matching with a replacement method (13, 14). Each alcoholic individual was paired with two non-alcoholic individuals who had the closest propensity score to minimize bias and improve the quality of matching; to compensate for the difficulty in identify confounding factors, indirect methods were used, and some of the alcoholic patients were paired with more than one control (60:128). Possible confounding factors or observable covariates were sex, age, obesity, vital state, pulmonary thromboembolism, ischemic temporal accident or stroke, confusion, diabetes, chronic obstructive pulmonary disease (COPD), arterial hypertension, anxiety, depression, asthma, liver disease (hepatitis or cirrhosis), smoking, human immunodeficiency virus (HIV), transplantation, cancer, cognitive impairment or dementia, and psychosis.

### EHR Semiautomatic Extracted Variables

The variables considered in the study were as follows: demographics (age and sex); personal health history (neuropsychiatric history and hepatic damage); severity risk for COVID-19 comorbidities (diabetes, arterial hypertension, obesity and overweight, chronic obstructive pulmonary disease); COVID-19 diagnosis (clinical diagnosis and PCR result); admission variables (length of stay and ICU admission).

### Outcomes

Vital state at hospital discharge, neuropsychiatric COVID-19 symptoms (psychosis, depression, anxiety, posttraumatic syndrome, cognitive impairment, confusion, transient ischemic attack, stroke, anosmia, ageusia).

### *Ad-hoc* Follow-Up Variables

Result variables: COVID-related chief complaint, vital state, length of stay, ICU admission, ICU length of stay, and complications. Adjustment variables: age, sex, personal history of alcoholism, diabetes, arterial hypertension, obesity and overweight, COPD, asthma, hepatitis, cirrhosis, toxic habits, smoking, human immunodeficiency virus (HIV), transplant, psychosis, depression, cognitive impairment or dementia, and anxiety. COVID-19 signs: levels of alkaline phosphatase (ALP), alanine transaminase (ALT), gamma-glutamyl transferase (GGT), reactive C-protein, D dimer, and fibrinogen at diagnosis. COVID-19 symptoms: pneumonia, type of pneumonia (unilateral, bilateral), fever, cough, asthenia, degree of asthenia, diarrhea, vomit, dyspnea, degree of dyspnea (low, moderate, severe), anosmia, ageusia, confusion, memory loss, psychotic crisis, delirium, depression, anxiety, sleep disorders, attention and concentration disorders, emotional lability, speech impairment, euphoria, aggressiveness, irritability, hallucinations, suicidal tendencies, posttraumatic syndrome, and consciousness level.

### Statistical Analysis

The variables were summarized by means and standard deviation or median (p50) and the interquartile range (p25-p75), according to the normality distribution. To check the normality distribution, the Shapiro–Wilk test was used. Qualitative variables were expressed in absolute numbers (number of cases) and in relative frequencies (percentage). To compare alcohol-related variables between groups, we used the Student's *T*-test or the non-parametric Mann–Whitney *U*-test, chi-square test (X^2^), or Fisher's exact test, depending on the nature of the variables.

A logistic regression model was created to study the association between possible risk factors for alcoholism adjusted for age, sex, obesity, cognitive impairment–dementia, HIV, smoking, transplantation, diabetes, anxiety, liver disease, depression, and psychosis. The results of the model are presented in the form of OR together with the 95% confidence interval (95% CI) (Table 1). The statistical study was completed with a multivariate analysis, considering both risk factors with a significant result in the univariate analysis and those that had some relevance (*p* < 0.1). The use of a selection by steps was used to highlight the most relevant factors, identify those significant sets among the possible variables, and avoid confusion in the model due to possible relationships between them since this method determines the significance of the effect of a variable in the presence of those already in the model. The Hosmer and Lemeshow test was used to evaluate the goodness of fit of the model.

**Table 1:** COHORT descriptive analysis.

	**Total *N =* 188**	**Non-alcoholic *N =* 128**	**Alcoholic *N =* 60**	***p*-value**
Age [mean (SD)]	60.45 (16.55)	59.93 (18.52)	61.56 (11.29)	0.53
Sex (% men)	134 (71.28%)	85 (66.41%)	49 (81.67%)	0.038
Non-COVID derivation (%)	39 (20.74%)	15 (11.71%)	24 (40.00%)	<0.001
COVID confirmed (%)	123 (65.43%)	72 (56.25%)	51 (85.00%)	<0.001
**Health outcomes**				
Vital state (%)	36 (19.15%)	23 (17.97%)	13 (21.67%)	0.56
Length of stay (IC95%)	5.00 (0.00–10.00)	2.00 (0.00–8.00)	9.00 (5.00–17.00)	<0.001
ICU admission	13 (6.91%)	4 (3.13%)	9 (15.00%)	0.005
ICU length of stay (95% CI)	0.00 (0.00–0.00)	0.00 (0.00–0.00)	0.00 (0.00–0.00)	<0.001
Need for respirator	26 (13.83%)	11 (8.59%)	15 (25.00%)	0.0049
PCR	101 (53.72%)	58 (45.31%)	43 (71.67%)	<0.001
Complications	42 (22.34%)	23 (17.97%)	19 (31.67%)	0.040
**Personal health history**				
Diabetes	47 (25.00%)	29 (22.66%)	18 (30.00%)	0.279
Arterial hypertension	75 (39.89%)	48 (37.50%)	27 (45.00%)	0.339
Obesity and overweight	28 (14.89%)	15 (11.72%)	13 (21.67%)	0.082
OCPD	29 (15.43%)	20 (15.63%)	9 (15.00%)	0.999
Asthma	13 (6.91%)	7 (5.47%)	6 (10.00%)	0.351
Hepatitis	31 (16.49%)	15 (11.72%)	16 (26.67%)	0.019
Cirrhosis	27 (14.36%)	6 (4.69%)	21 (35.00%)	<0.001
Drug addiction	15 (7.98%)	4 (3.13%)	11 (18.33%)	<0.001
Smoker	44 (23.40%)	22 (17.19%)	22 (36.67%)	<0.001
Ex-smoker	21 (11.17%)	8 (6.25%)	13 (21.67%)	
HIV	6 (3.19%)	3 (2.34%)	3 (5.00%)	0.389
Transplant	13 (6.91%)	7 (5.47%)	6 (10.00%)	0.348
Psychosis	5 (2.66%)	3 (2.34%)	2 (3.33%)	0.651
Depression	38 (20.21%)	20 (15.63%)	18 (30.00%)	0.031
Suicidal attempts	6 (3.19%)	2 (1.56%)	4 (6.67%)	0.083
Cognitive impairment	20 (10.64%)	10 (7.81%)	10 (16.67%)	0.078
Anxiety	27 (14.36%)	20 (15.63%)	7 (11.67%)	0.509
**Laboratory parameters**				
AST	35.0 (24.0–51.0)	33.0 (24.0–44.0)	42.0 (26.0–70.0)	0.066
ALT	66.0 (27.0–131.5)	50.0 (24.0–99.0)	115.0 (49.0–255.0)	<0.001
Gama GT	133.53 (278.5)	72.74 (72.49)	236.33 (430.18)	<0.001
C-reactive protein	5.60 (1.43–12.82)	6.44 (1.34–12.49)	4.70 (1.62–16.56)	0.789
Fibrinogen	658.09 (207.17)	658.35 (194.77)	657.67 (229.24)	0.989
MCV	91.35 (7.03)	91.13 (6.57)	91.74 (7.83)	0.609
D-dimer	786 (414–1,463)	669 (381–1,488)	992 (600–1,463)	0.390
**COVID-19 debut symptoms**				
Pneumonia (yes/no)	103 (54.79%)	65 (50.78%)	38 (63.33%)	0.119
Unilateral pneumonia	22 (11.70%)	13 (10.16%)	9 (15.00%)	0.219
Bilateral pneumonia	81 (43.09%)	52 (40.63%)	29 (48.33%)	
Fever	114 (60.64%)	75 (58.59%)	39 (65.00%)	0.431
Cough	94 (50.00%)	64 (50.00%)	30 (50.00%)	1.000
Asthenia	41 (21.81%)	30 (23.44%)	11 (18.33%)	0.459
Diarrhea	46 (24.47%)	30 (23.44%)	16 (26.67%)	0.719
Vomits	15 (7.98%)	11 (8.59%)	4 (6.67%)	0.782
Dyspnea	86 (45.74%)	52 (40.63%)	34 (56.67%)	0.043
Anosmia	15 (7.98%)	13 (10.16%)	2 (3.33%)	0.149
Ageusia	15 (7.98%)	12 (9.38%)	3 (5.00%)	0.389
Anosmia and Ageusia	8 (4.26%)	6 (4.69%)	2 (3.33%)	1.000
Confusion	22 (11.70%)	9 (7.03%)	13 (21.67%)	0.006
Memory loss	7 (3.72%)	2 (1.56%)	5 (8.33%)	0.035
Psychotic crisis	2 (1.06%)	0 (0.00%)	2 (3.33%)	0.101
Depression	8 (4.26%)	5 (3.91%)	3 (5.00%)	0.711
Anxiety	12 (6.38%)	7 (5.47%)	5 (8.33%)	0.531
Sleeping problems	11 (5.85%)	7 (5.47%)	4 (6.67%)	0.749
Attention problems	12 (6.38%)	7 (5.47%)	5 (8.33%)	0.529
Emotional lability	8 (4.26%)	3 (2.34%)	5 (8.33%)	0.111
Disorders of consciousness	21 (11.17%)	12 (9.38%)	9 (15.00%)	0.319
Speech impairment	6 (3.19%)	5 (3.91%)	1 (1.67%)	0.67
Aggressiveness	3 (1.60%)	0 (0.00%)	3 (5.00%)	0.031
Irritability	2 (1.06%)	0 (0.00%)	2 (3.33%)	0.100
Hallucinations	4 (2.13%)	1 (0.78%)	3 (5.00%)	0.097
Other NP symptoms	19 (10.11%)	7 (5.47%)	12 (20.00%)	0.004
At least one NP symptom	51 (27.13%)	25 (19.53%)	26 (43.33%)	0.001
Number of NP symptoms (median)	0.00 (0.00–1.00)	0.00 (0.00–0.00)	0.00 (0.00–2.00)	<0.001

*Frequency and statistical significance between variables in patients with any annotation in their medical record of alcohol abuse (alcoholic) vs. patients without a reference to alcohol abuse (non-alcoholic)*.

*NP, neuropsychiatric; MCV, mean corpuscular volume; COPD, chronic obstructive pulmonary disease; HIV, human immunodeficiency virus; ICU, intensive care unit; GGT, gamma-glutamyl transferase; GOT (AST), glutamyl oxaloacetic transaminase/aspartate aminotransferase; non-COVID derivation, patient who seek medical attention due to symptoms initially unrelated to COVID-19*.

All analysis were performed using the STATA version 16 statistical software. In all cases, the level of confidence was 95%, considering that there was statistical significance when *p* ≤ 0.05.

## Results

**Figure 1:**
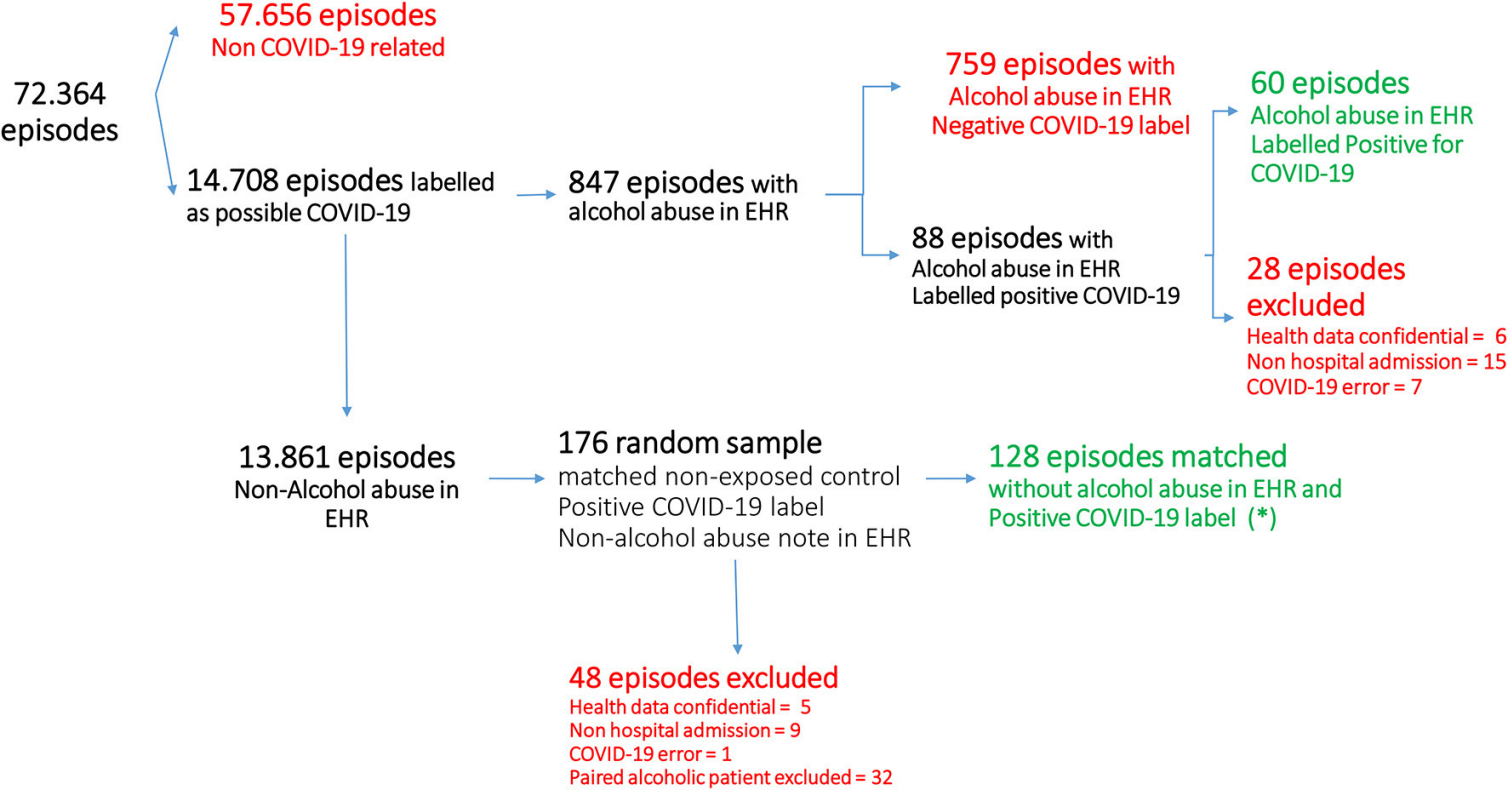
Patients flow and final cohort identification. *During the matching process, to compensate for the difficulty in identify cofounding factors, some of the alcoholic patients were paired with more than two controls.

During the study period, the Hospital Universitario 12 de Octubre offered health assistance (Figure 1) in 72,364 episodes to patients either at the emergency department, as inpatients or as a reference laboratory for the PCR technique from Primary Care. Fourteen thousand seven hundred eight had a diagnosis of COVID-19; 847 had at least one diagnosis or reference to a personal history of alcoholism (1.17% of the total episodes) of whom 88 had COVID-19 (10.39% of the alcoholic patients; 0.6% of the COVID-19 patients). Table 1 summarizes the descriptive analysis of demographic-related variables. The cohort of patients diagnosed with alcohol abuse had a higher proportion of male patients (*p* = 0.032).

Twenty-eight patients who met the exclusion criteria were removed from the cohort. Six of the patients were hospital workers, and their data were confidential occupational health data. Fifteen were misclassified since they had no hospital admission, having only a positive PCR demanded from the Primary Care. Lately, seven patients were misclassified as “confirmed COVID-19,” but they had no COVID-19 diagnosis or it was finally ruled out. Thus, the final cohort was composed of 60 patients with a confirmed personal record of alcoholism, a diagnosis of COVID-19, and an EHR episode registered as “alcoholic.” Alcoholic patients were paired 2:1 with 128 non-alcoholic patients. We considered non-alcoholic patients as those who had an explicit comment of “no alcohol consumption” or the absence of a diagnosis of alcohol abuse in the medical records.

In the descriptive analysis of the historic cohorts (see Table 1), there were statistically significant differences in the length of inpatient stay (*p* < 0.001), being longer for alcoholic patients with a mean of 9 days compared to non-alcoholics in which the mean was 5 days. There were also differences in the frequency of ICU admission (*p* = 0.005) and in the length of stay in ICU (*p* < 0.001), as well as in the need of respirator (*p* = 0.0049). However, it is important to highlight that the variables of cirrhosis (*p* < 0.001) and other toxic habits such as smoking (*p* < 0.001) and drug addiction (*p* < 0.001) were significantly higher in the alcoholic cohort, which could be related to the severity of the disease.

As for the COVID-19 symptoms, the univariate analysis showed differences in several result variables. The final multivariable model showed no significant differences for respiratory or digestive symptomatology with the exception of dyspnea (*p* = 0.043). There were, however, statistically significant differences in the neuropsychiatric symptoms (*p* = 0.001); statistical significance was reached for confusion (*p* = 0.006), memory loss (*p* = 0.035), violent behavior (*p* = 0.031) and other neuropsychiatric symptoms (*p* < 0.001).

The variable “other neuropsychiatric symptoms” was diverse, including pain (*n* = 4), stroke (*n* = 2), agitation (*n* = 2), confusion and disorientation (*n* = 2), hepatic encephalopathy (*n* = 2), weakness and walking impairment (*n* = 3), bradypsychia (*n* = 2), dysarthria (*n* = 2), Guillain-Barre syndrome (*n* = 1), hemiparesis (*n* = 1), panic attack (*n* = 1), dementia (*n* = 1), diplopia (*n* = 1), and syncope (*n* = 1). Five of the patients showed more than one symptoms. The neuropsychiatric symptoms (NP symptoms) variable included all the neuropsychiatric symptoms but not ageusia and anosmia, which were analyzed separately.

One hundred one (53.72%) patients of the global cohort were diagnosed with COVID-19 having a supporting positive PCR test for SARS-CoV-2, while the other 87 patients were diagnosed as COVID-19 without this diagnosis test, receiving only a clinical diagnosis, most probably due to the initial lack of test supplies. Patients with a personal history of alcohol abuse were more frequently tested as positive for SARS-CoV-2 than patients without personal records of alcoholism (*p* = 0.003) and more frequently (40.00 vs. 11.71%) requested medical assistance in the hospital setting with the suspicion of COVID-19 (*p* < 0.001).

Adjustment was incomplete due to certain characteristics of alcoholism, such as its association to other toxic habits, for instance drug abuse and smoking (*p* < 0.001), more frequent than in non-alcoholic patients, and the effects of alcohol over the liver function that can lead to cirrhosis (*p* < 0.001) and hepatitis (*p* = 0.025). In addition, depression (*p* = 0.031) was not completely paired in the non-alcoholic population. Thus, the analytical variables measuring liver function (ALT and GGT) showed significant differences (*p* < 0.001) between alcoholic and non-alcoholic patients. However, the median values in these variables were in the pathological range in both cohorts; besides, depression as a COVID-19 symptom had no significant differences between both cohorts.

In the univariate analysis of the paired cohorts, patients with a personal history of active alcohol abuse were 8% more likely to extend their length of stay for 1 day (CI_95%_ = 1.04–1.12) and 15% more likely to extend their ICU length of stay (CI_95%_ = 1.01–1.30). They were 5.47 times more at risk of needing ICU admission (CI_95%_ = 1.61–18.57) and 3.54 times (CI_95%_ = 1.51–8.30) more at risk of requiring a respirator. As for the symptoms of COVID-19, patients with a personal history of alcohol abuse showed 91% more frequency of dyspnea (CI_95%_ = 1.03–3.55), they were 3.15 times more at risk of exhibiting at least one neuropsychiatric symptom (CI_95%_ = 1.61–6.17), and they reached statistically significant differences in the number of NP symptoms developed during the COVID-19 infection. In the final model for the multivariate analysis (see Table 2), patients with a personal history of alcohol abuse were 2.38 times more at risk of developing at least one neuropsychiatric symptom (CI_95%_ = 1.01–5.59). Through the Hosmer and Lemeshow test, we can verify that the goodness-of-fit test in the proposed multivariate model (Annex 1) is considered a good fit since the value of the Cg statistic was 0.8324.

**Table 2:** Logistic regression model, odds ratios in univariate, and multivariate analysis.

	***p*-value**	**OR (95% CI)**	Multivariate analysis (^*^) OR (95% CI)
Length of stay	<0.001	1.08 (1.04–1.12)	1.07 (1.03–1.12)
ICU admission	0.029	1.15 (1.01–1.30)	
AST	0.045	1.01 (1.00–1.02)	
GGT	<0.001	1.01 (1.00–1.01)	1.01 (1.00–1.01)
C-reactive protein	0.994	0.99 (0.96–1.03)	
Fibrinogen	0.987	0.99 (0.99–1.00)	
MCV	0.611	1.01 (0.96–1.06)	
D-dimer	0.350	0.99 (0.99–1.00)	
Number of NP symptoms	0.009	1.31 (1.07–1.61)	
Death	0.549	0.79 (0.37–1.69)	
ICU admission	0.006	5.47 (1.61–18.57)	
Need of respirator	0.004	3.54 (1.51–8.30)	
PCR	0.001	3.05 (1.58–5.91)	
Complications	0.038	2.11 (1.04–4.29)	
COPD	0.912	0.95 (0.40–2.24)	
Asthma	0.260	1.92 (0.62–5.98)	
Hepatitis	0.012	2.74 (1.25–6.01)	2.46 (0.85–7.08)
Drug addiction	0.001	6.96 (2.11–22.90)	
Suicidal attempts	0.088	4.50 (0.80–25.29)	
COVID	<0.001	4.41 (2.00–9.71)	3.22 (1.02–10.09)
Pneumonia	0.180	1.67 (0.89–3.14)	
Unilateral pneumonia	0.171	1.98 (0.74–5.27)	
Bilateral pneumonia	0.168	1.59 (0.82–3.10)	
Fever	0.403	1.31 (069–2.48)	
Cough	0.999	1.00 (0.54–1.85)	
Asthenia	0.431	0.73 (0.34–1.58)	
Diarrhea	0.631	1.19 (0.49–2.40)	
Vomits	0.650	0.76 (0.23–2.49)	
Dyspnea	0.041	1.91 (1.03–3.55)	
Anosmia and ageusia	0.670	0.70 (0.14–3.58)	
NP symptoms	0.001	3.15 (1.61–6.17)	2.38 (1.01–5.59)

*NP, neuropsychiatric; MCV, mean corpuscular volume; COPD, chronic obstructive pulmonary disease; ICU, Intensive Care Unit; GGT, gamma-glutamyl transferase; GOT (AST), glutamyl oxaloacetic transaminase/aspartate aminotransferase*.

* *Drug addiction was excluded due to the ample range of the CI*.

## Discussion

The most relevant results of our study indicate that patients with COVID-19 infection and alcohol abuse had more complications as suggested by longer lengths of stays, greater need for ICU and ventilator support, and significantly more neuropsychiatric complications such as the presence of confusion, memory deficits, violent behavior, and other neuropsychiatric symptoms. However important for prognosis and management of patients, alcohol abuse conditions are neither properly identify on the EHR nor systematically screened while caring for a patient; and this can have important impact on the patient's healthcare.

Regarding COVID-19 complications, our findings are consistent with those of other studies that have also found that high-dose alcohol consumption increases the risk for ARDS (9), ICU admission, and longer hospital lengths of stay (10). Chronic alcohol consumption can induce ciliary dysfunction in the respiratory tract that reduces the capacity for bacterial and viral clearance (15).

Possibly the most suggestive finding in our study is the correlation between a personal history of alcoholism and neuropsychiatric complications, as evidenced by the presence of significant symptoms in that group such as confusion, memory loss, violent behavior, and other neuropsychiatric manifestations. These manifestations may reflect the greater severity of the disease, and, in fact, in a study that included patients admitted to ICU, up to 84% presented neurological complications, indicating that these were more frequent in the most severe cases (16). It could also be due to the presence of the virus in the central nervous system (CNS), as it has been pointed out in a recent review of the neurological complications of COVID-19 (17).

There are multiple possible routes of entry of the virus into the CNS: through the olfactory bulb, through the blood–brain barrier (BBB) (18), through infected leukocytes, or through axonal transport in peripheral nerves (19). The enzyme angiotensin-converting enzyme 2 (ACE2), through which the virus enters into the cell, is found in the cerebral vascular endothelium (17). COVID infection can damage endothelial cells and activate proinflammatory and thrombotic pathways (17). Thus, the most severe symptoms may also be a consequence of the cytokine storm (16).

One of the plausible hypothesis to explain the increased CNS vulnerability to the COVID infection in patients with a personal history of alcohol abuse is that ethanol is able to interact forcefully at different levels, acting on both natural or innate immunity [phagocytosis, natural killer (NK) cells, complement] and on specific or acquired immunity (20, 21). In particular, the activity of NK cells is altered by the following actions of ethanol: interference of the bond between NK and the target cells, modification of the production and use of some cytokines, alteration of cytolytic activity, alteration of signal transduction, and a direct effect on the neuro-endocrine system (22). Another entry pathway could be related to an increased permeability of the BBB (23, 24), which would favor the proinflammatory cascade caused by the virus.

As for the strengths and limitations of the study, one of the main issues observed during the pandemic is the insufficient quality of clinical and epidemiological data to design observational studies and to elaborate hypothesis for future researches. Another key difficulty is to identify patients who meet clinical characteristics of interest, other than COVID-19-related information (currently well-recorded). Automatic extraction of information from EHR could be of great help in a pandemic. This study has been supported by real world data (RWD) semiautomatically extracted from EHR and compared with the current gold standard for retrospective studies, the manual EHR review. The iterative approach for patient cohort identification has been very specific in the variable's identification even though not very sensitive (<2% of patients with a personal record of alcohol abuse were identified, although within inpatients, it was expected to be identified around 20%). In this way, one supposedly alcoholic patient was considered as non-alcoholic and three non-alcoholic patients were identified as alcoholic by the reviewers. Thus, 98.3% of the patients identified as patients with a personal history of alcohol abuse were confirmed as so by individual and manual review of the health records of every single patient (gold standard), and 97.6% of the patients identified as non-alcoholic were confirmed as so by the gold standard.

Despite this strength, several limitations should be taken into account. First, identifying alcoholism exposure was done following medical records of EHR and not any other objective measures. For this reason, there could be a selection bias since alcoholism, among other substance abuse conditions, is a stigmatized disease that carry psychological and physical suffering to the patients who could minimize or deny their alcohol consumption; in addition, because alcohol consumption is considered part of a cultural legacy within our social context, it could be not systematically recorded in the patient's medical records, underdiagnosing alcohol abuse. Moreover, the chances of being codified as an alcoholic are higher when associating pathologies such as cirrhosis or a severe dependence. Finally, drug abuse and smoking habit are systematically explored in patients identified as alcoholics but not in non-alcoholics, and therefore, the significant differences observed in these variables could be due to an appropriate diagnosis in alcoholic and ex-alcoholic patients but a misdiagnosis in non-alcoholic patients.

On the other hand, medical history should have been structured in the “personal health history” section of EHR, and it has not, and in this way, the semiautomatic identification of variables could have been mistaken. Ideally, a multicentric observational prospective cohort study including the variables from our database and the systematic recording of toxic habits in all COVID-19 inpatients could greatly reinforce our conclusions regarding the impact of alcohol abuse in COVID-19 neuropsychiatric symptoms and prognosis. In fact, alcohol consumption has an important effect on the central nervous system and on mental health and, in COVID-19 patients, seems to increment significantly the probabilities of suffering at least one neuropsychiatric symptoms and frequently more than one. Unfortunately, the insufficient size of the cohort and the intrinsic limitations of this study do not allow a stronger assessment; however, it is a research topic worth exploring in depth.

In conclusion, considering the use and abuse of alcohol that is occurring in the general population as a consequence of the pandemic, it would be advisable to warn of the negative consequences that these habits may have on the complications of COVID-19 infected people.

Clinicians should systematically assess history of alcohol issues and drinking habits in all patients, especially for those who seek medical advice regarding COVID-19 infection in order to prevent symptoms severity and complications that results from this association.

Information regarding personal health records should be recorded in a structured field of EHR to facilitate the automatic extraction of data for observational studies in real time, useful to evaluate the decision-making process in a dynamic context.

## Data Availability Statement

The original contributions presented in the study are included in the article/Supplementary Material, further inquiries can be directed to the corresponding author/s.

## Ethics Statement

Ethical review and approval was not required for the study on human participants in accordance with the local legislation and institutional requirements. Written informed consent for participation was not required for this study in accordance with the national legislation and the institutional requirements.

## Author Contributions

CV, FA, and GR contributed in the study desing, data recording, manuscript writing, and scientific discussion. CM-A did the statistical analysis and patient matching and propensity score adjustment. CC, AJ, ME, IR, LS, and ÁC participated in the data recording and colaborated in science discussion. BB and MM participated in the english translation. All authors contributed to the article and approved the submitted version.

## Conflict of Interest

The authors declare that the research was conducted in the absence of any commercial or financial relationships that could be construed as a potential conflict of interest.
