# Supplementary material for: COVID-19-Related Neuropsychiatric Symptoms in Patients With Alcohol Abuse Conditions During the SARS-CoV-2 Pandemic: A Retrospective Cohort Study Using Real World Data From Electronic Health Records of a Tertiary Hospital
Source: Front Neurol. 2021 Mar 3;12:630566. doi: 10.3389/fneur.2021.630566 (PMC7966461; doi:10.3389/fneur.2021.630566)
Supplement: Supplementary file 1 [file Data_Sheet_1.docx]

ANNEXE 1: LOGISTIC REGRESSION MODEL

**Final model**

Logistic regression Number of obs = 148

LR chi2(5) = 52.29

Prob > chi2 = 0.0000

Log likelihood = -71.505149 Pseudo R2 = 0.2678

| Alcoholismo_NVO | Odds Ratio | Std. Err. | z | P>\|z\| | [95% Conf. Interval] | |
| --- | --- | --- | --- | --- | --- | --- |
| Esta0ciahospitalaria | 1.07376 | 0.02417 | 3.16 | 0.002 | 1.02742 | 1.12219 |
| GamaGT | 1.00735 | 0.00207 | 3.56 | 0.000 | 1.00330 | 1.01141 |
| _IHepatitis_1 | 2.45780 | 1.32694 | 1.67 | 0.096 | 0.85308 | 7.08112 |
| _ICOVID_1 | 3.21820 | 1.87724 | 2.00 | 0.045 | 1.02586 | 1.00957 |
| _INPSQ_1 | 2.37644 | 1.03769 | 1.98 | 0.047 | 1.00982 | 5.59254 |
| _cons | 0.02958 | 0.02042 | -5.10 | 0.000 | 0.00764 | 0.11448 |

Note: _cons estimates baseline odds.

**Covariance matrix of coefficients of logit model**

|  | Alcoholismo_NVO |  |  |  |  |  |
| --- | --- | --- | --- | --- | --- | --- |
| e(V) | Esta0ciahospitalaria | GamaGT | IHepatitis_1 | _ICOVID_1 | _INPSQ_1 | _cons |
| Alcoholismo_NVO |  |  |  |  |  |  |
| Esta0ciahospitalaria | .00050654 |  |  |  |  |  |
| GamaGT | 0.004001 | 0.004219 |  |  |  |  |
| IHepatitis_1 | .00205834 | -.00005935 | .29148104 |  |  |  |
| _ICOVID_1 | -.00104862 | .00008727 | .01979201 | .34026278 |  |  |
| _INPSQ_1 | .00019124 | .00005621 | .02449887 | .04421634 | .19066782 |  |
| _cons | -.00516875 | -.00057186 | -.09547268 | -.3061396 | -.12001266 | .47682329 |

**Logistic model for Alcoholismo_NVO. goodness-of-fit test**

(Table collapsed on quantiles of estimated probabilities)

number of observations = 148

number of groups = 10

Hosmer-Lemeshow chi2(8) = 2.54

Prob > chi2 = 0.9597

**Area under ROC curve (Statistical index C)**

number of observations = 148

area under ROC curve = 0.8324
